# The influence of religiosity on food choice among British Muslims: A qualitative study

**DOI:** 10.1177/02601060241244883

**Published:** 2024-04-03

**Authors:** Umama Owais, Riya Patel, Sally Abbott

**Affiliations:** 1 School of Health and Life Sciences, 2706 Coventry University, Coventry, UK; 2 Research Centre for Healthcare and Communities, Institute of Health and Wellbeing, Coventry, UK

**Keywords:** Islam, Muslim, food choice, religiosity, qualitative

## Abstract

**Background:** Religiosity is known to have a socio-cultural influence on food choice. However, to date, research exploring the influence of Islam on food selection has almost exclusively focused on fasting during Ramadan and has not explored the influences of Islam on everyday food choices among Muslim people. **Aim:** This qualitative study explored the influence of Islamic religiosity on everyday food choices among Muslim people. **Methods:** Thirty-two adult participants residing in the United Kingdom (*n* = 16 faith leaders and *n* = 16 lay Muslim people) were recruited from three Sunni mosques, and data was collected using semi-structured interviews. The data was analysed using reflexive thematic analysis and a constant comparison method was applied to draw out similarities and differences between faith leaders and lay Muslim people. **Results:** The results revealed that Islamic religiosity had an influence over food choice with two main overarching themes 1) Demonstrating religious obedience through food choices and, 2) Spheres of influence on food choice; and five sub-themes 1a) Trusting in familiar food providers, 1b) Verification of halal authenticity, 1c) Seeking purity within food, 2a) The Prophet Muhammed (Peace Be Upon Him) as a role model for food choice and, 2b) Islamic jurisprudence. **Conclusion:** These findings provide important insights into the influence of Islam on food choice and could be used support the design of faith-informed dietary interventions among Muslim people. Further research is required to examine the role of faith-informed dietary intervention in the Muslim community.

## Introduction

Health beliefs and values vary between cultures and religions and all healthcare professionals should be able to provide culturally responsive care. Culturally responsive care is tailored to the needs of the individual patient and considers the unique cultural, spiritual and religious factors that influence their health (Office for Health Improvement and Disparities, 2021). Healthcare professionals must learn about cultural influences on food and nutrition to provide dietary education that is specific to the patient and is delivered in a culturally sensitive manner (Ring et al., 2023).

Understanding the role of food in religious practices can assist healthcare professionals and wider society to respect and respond to the needs of people from religious communities (Chouraqui et al., 2021). Religion can define food practices through various rules and symbols in religious teachings. As a result, certain food choices can be a symbolic act or part of a ritual to demonstrate faith and enhance feelings of belongingness (Monterrosa et al., 2020).

Muslims, the people who follow Islam's teachings, comprise the second-largest religious community in the world, with nearly a quarter (24.9%) of the world population identifying as Muslim in 2020 (Pew Research Center, 2022). Islam is also the second-largest practiced religion in the United Kingdom, with a population of 3.87 million Muslim people living in England and Wales, comprising 6.5% of the population (Office for National Statistics, 2023).

As religiosity has a socio-cultural influence on food choice, religious teachings may be used as a powerful delivery channel to promote health through dietary behaviours. Understanding Islamic perspectives on diet will assist healthcare professionals to deliver appropriate nutrition care in a culturally sensitive manner (Ring et al., 2023). The religion of Islam places great emphasis on food consumption, dietary habits and food etiquette. The Holy Quran, a prominent Islamic scripture, contains over 250 verses about nutritional concepts, including avoiding eating and drinking haram (unlawful) foods (e.g. pork and its derivatives and alcohol), while halal (permissible) and tayyib (pure) foods are encouraged (Tarighat-Esfanjani and Namazi, 2016).

Despite the centrality of food in Islamic scripture, research on religion and food habits in Islamic culture is lacking (Norman, 2012). The published qualitative research on nutritional behaviours in Muslim populations has, to date, focused on the practice of fasting during Ramadan (Alghafli et al. 2019; Hasan et al., 2021), and on populations with specific health conditions, such as type 2 diabetes (Bouchareb et al., 2022; Lee et al., 2017) and chronic kidney disease (Adanan et al., 2021).

However, there is an absence of qualitative research undertaken in the UK on the influence of Islam on everyday dietary behaviour, and there is no literature comparing faith leader and lay Muslim perspectives. Therefore, the aim of this study was to explore the influence of Islamic religiosity on food choice among British Muslim people.

## Materials and methods

This study is reported in accordance with the ‘Consolidated criteria for reporting qualitative research’ checklist (Tong et al., 2007).

### Study design

The authors conducted an exploratory qualitative study to explore the influence of Islamic religiosity on experiences of food choice, seeking the perspectives of both faith leaders and lay Muslims. Since the literature on this topic is limited and not well established, a qualitative approach provided the freedom and flexibility required to investigate this phenomenon (Leedy and Ormrod, 2015).

### Researcher positionality

In this study, the influence of the lead author's (UO) religious affiliation with Islam and experiences of being a Muslim of Pakistani ethnicity has been acknowledged throughout the research process, including their role as an interviewer, by exercising reflexivity. The positionality of the co-authors (SA a dietitian of White ethnicity and an atheist; RP a qualitative researcher of British Indian ethnicity and a practicing Christian) is also considered, regarding their involvement with data analysis and interpretation of findings.

### Participants

Practicing Muslims aged 18 and over, fluent English language speakers, and either faith leaders (e.g. Imams, Islamic theologists etc.) or practicing lay Muslims were eligible to take part in the study. A purposive sample of faith leaders and lay Muslims was recruited via flyers at three Sunni mosques (West Midlands n = 1; West Sussex n = 2) to ensure equal numbers of faith leaders and lay Muslims in the study. Recruitment took place between July 2021 and October 2022. Participants were encouraged to share the study advertisement with other eligible potential participants via snowball sampling methods.

### Data collection

At the point of consent, 34 participants provided their demographic details, including age, gender, ethnicity and vocation. Two participants were not responsive to invitations to arrange interviews. Semi-structured interviews (duration ranging from 24 min to 90 min) were then conducted by one interviewer (UO) over Zoom or Microsoft Teams using an interview topic guide. The topic guide was informed by the study objectives and piloted with one Muslim person, which lead to some minor modifications to the wording of the questions. All individuals involved in the study gave their written informed consent before arranging an interview. All interviews were audio-recorded and transcribed verbatim by the lead researcher (UO) to ensure authenticity (Lingard, 2019). The transcripts of all the participants were anonymised to ensure confidentiality.

### Data analysis

Transcripts were read and re-read to facilitate the researcher's immersion in the data. Reflexive thematic analysis (Braun and Clarke, 2006) was used to analyse the data. Transcripts were first coded individually, where themes related to food choices were developed. Following this, a constant comparative method was performed where similarities and differences between the two participant groups (faith leaders and lay Muslims) were identified (Maykut and Morehouse, 2005). This was an iterative process, where discussions within the research team further refined and developed the themes. Overarching themes and sub-themes that captured these similarities and differences were then developed for the final analysis. UO, SA and RP engaged in peer review at the data interpretation stage, to enhance credibility.

### Ethical approval

Ethical approval was granted by Coventry University Ethics Committee (P121546).

## Results

### Participant characteristics

Thirty-two Sunni Muslim participants (n = 16 faith leaders n = 16 lay Muslims) were interviewed from four regions within the UK: West Midlands (n = 13), London (n = 10), West Sussex (n = 8) and Greater Manchester (n = 1). Most participants (28/32; 87.5%) identified as South Asian ethnicity. Most lay Muslims were younger (n = 13 aged 20–29 years old) than faith leaders (n = 3 aged 20–29 years old). See Table 1 for details of demographics at the participant level.

**Table 1.** Participant characteristics.

Participant	Gender	Age	Faith leader	Congregant	Ethnicity
1	Male	23		✓	Pakistani
2	Male	53	✓		British-Indian
3	Female	23		✓	British-Indian
4	Female	40	✓		British-Indian
5	Female	29		✓	British-Indian
6	Male	28	✓		British-Pakistani
7	Male	39		✓	British-Pakistani
8	Male	34	✓		Sri Lankan
9	Female	23		✓	British-Pakistani
10	Female	36	✓		British-Pakistani
11	Female	20		✓	British-Bangladeshi
12	Female	20		✓	British-Bangladeshi
13	Female	27		✓	British-Bangladeshi
14	Female	NR		✓	Arab
15	Female	47	✓		British- Pakistani
16	Female	21		✓	Arab
17	Male	42	✓		British-Pakistani
18	Male	27	✓		British-Pakistani
19	Male	39	✓		Indonesian
20	Male	41		✓	Indonesian
21	Female	26		✓	Indian Mauritian
22	Male	22		✓	British-Pakistani
23	Female	39	✓		British-Indian
24	Female	23	✓		British-Afghan
25	Male	30	✓		British-Indian
26	Male	40	✓		British-Bangladeshi
27	Male	47	✓		British-Indian
28	Female	34	✓		British-Indian
29	Female	44	✓		British-Bangladeshi
30	Male	20		✓	British-Sri Lankan
31	Female	20		✓	British-Sri Lankan
32	Female	25		✓	British Other Asian

*Note:* NR: not recorded.

### Themes and sub-themes

Two overarching themes with a total of five sub-themes were developed and are presented below, with accompanying illustrative participant quotations.

#### Theme 1: Demonstrating religious obedience through food choices

Participants stated their purpose in life is to please God by only consuming foods that are permissible in Islam, to demonstrate their devotion to God, the Qur’anic teachings and the Sunnah of the Prophet Muhammad, peace be upon him (PBUH).

Participants spoke of ‘wisdom’ (hikmah) in what has been ordained for them to abide by. The dietary rules of halal and haram in Islam were perceived as being for their own ‘benefit’ and thus food selection was not actually perceived as a food ‘choice’, but as a required act of obedience to God. Participants were clear in their understanding of what foods are haram and halal. Some shared Qur’anic verses that spoke of God promising them a guaranteed ‘ajar’ (reward) in this life and the hereafter if they make food choices in line with Islamic dietary rules.

The physical environment posed challenges for Muslim people to be able to identify, and therefore to consume, food that was truly halal. This is reflected in the sub-themes of ‘trusting in familiar food providers’, ‘verification of halal authenticity’ and ‘seeking purity within food’; which illustrate how Islamic teachings influence the decision-making processes surrounding food selection.

##### Sub-theme: Trusting in familiar food providers

There was cautiousness around the authenticity of foods claiming to be ‘halal’, especially when it came to meat and animal derivative products. Decisions around sourcing, purchasing and consuming meat were based on familiarity with, and trust in, reputable individual(s) and/or small businesses from within their own communities.

There was credibility in the ‘word’ of the individual(s) supplying the food if they were a lay Muslim of a mosque within their locality, and someone whom others from their Islamic community endorsed: ‘*…if I know the person and I have seen he is a worshipper and is a good person, he won't lie on that matter. Then of course, I will consume if he says it is halal, but if I don't know the person, then I will go with a certificate, or evidence*’ (Participant 17, faith leader).

Trusting in familiarity was further demonstrated when participants discussed UK supermarket chains (e.g. Asda, Sainsbury's). Participants were less trusting that the food was authentically halal because they were unable to identify information about the meat in the same way they would from a known independent Halal butcher. Supermarket workers were not perceived to have intimate knowledge of the meat supply chain, in comparison to a local halal butcher, which resulted in doubt when buying meat from UK supermarket chains: ‘*You know who these people are, you know the certificates, you can even ask them where they get their meat from, and they're cutting the meat in front of you. Some of the brands that the big grocery stores have for halal food are a bit questionable*’ (Participant 22, lay Muslim).

Greater trust in food prepared in familiar settings was also demonstrated with Muslim people preferring home food as their ‘trusted source’ due to its ‘convenience’ and ‘taste’; and would generally elect to choose home-cooked traditional meals over takeaways: ‘*I tend to eat a lot of food at home, just because it's easier and the food is better anyway. Generally, I also take food from home to places like work*’ (Participant 22, lay Muslim); ‘*…the way you consume your food, where it comes from, and what you particularly choose plays a vital role when it comes to the spiritual connection with Allah. So that's my reason for strongly picking the home cooked food as my trusted source*’ (Participant 6, faith leader).

Reassurance was sought by Muslim people to ensure that the food they are consuming is not contaminated with impermissible ingredient(s) and/or products. Participants shared that they believed that the food they consumed ultimately influenced their spirituality and connection with God, and consumption of food that is haram would have a negative impact on their ibadah (worship). ‘*I’m very vigilant because I know when I neglect or consume [food] that is unlawful it will impact my worship. Everything we eat and drink is ibadah and a kind of worship. Therefore, a prerequisite of acceptance is purely khaalis [pure] food without any contamination of unlawful things*’ (Participant 20, lay Muslim).

There was a feeling of insecurity around the consumption of food when travelling outside of their local community, to the extent that Muslim people may avoid eating meat products altogether: ‘*When I am out from my locality, I actively refrain from eating anything. I'd go for something vegetarian if I wasn't confident that the place was serving halal*’ (Participant 26, faith leader).

Participants reflected a preference to travelling to Muslim countries for vacations, as it was perceived that meat and animal derivatives in Muslim countries would be universally halal, which provided an air of convenience and comfort when it came to food selection: ‘*Whenever we go to a new town, we always make sure that there's halal food options. When you go abroad to a Muslim country, everything is halal*’ (Participant 12, lay Muslim); ‘*We enjoy going to countries where everything is halal. … [because] sometimes [in the UK] you have to actually question whether it is halal even though it's got a sign, it can be difficult, especially if you're going out of town to an area that you’re not familiar with. So, it's a lot of research beforehand, to be able to see if those places are suited to our diet*’ (Participant 5, lay Muslim).

##### Sub-theme: Verification of halal authenticity

When deciding whether food was ‘worthy’ of consumption, Muslim people sought reassurance from recognised certification. Most faith leaders sought verification by trusting and accepting only Halal Monitoring Committee (HMC) certified foods: ‘*HMC just gives you the peace of mind that it (meat) is slaughtered in the correct manner*.’ (Participant 28, faith leader). HMC certified meat was considered by participants to be ‘the gold standard’ of halal certification, whereas emblems at catering venues solely denoting ‘halal’ were perceived to have less validity.

Faith leaders showed a stronger sense of determining the authenticity of halal food due to their knowledge and responsibility as a learned individual within the community. For faith leaders, in the absence of HMC certification and where there were ‘warning signs’ of the venue itself being truly halal, they applied a proactive approach and sought further validation of the claims of ‘halal’ food: ‘*…if I have been told by our people, that this place is reputable, I still tend to be sceptical, but I will go and ask the owners or the people that are working inside. That would also help me influence my decision before I will eat. [If] he knows where he got his meat from…and he understands what stunned meat is and what un-stunned meat is, ‘yes’, I would then eat*’ (Participant 25, faith leader). ‘Warning signs’ included where the venue served halal food and drinks alongside haram food and/or drinks (e.g. catering venues that also served alcohol). In such instances, faith leaders were sceptical, and they asked questions about cross contamination, requested proof of certificates and/or names of meat suppliers.

On the other hand, lay Muslim participants did not always seek further validation if they were unconvinced over the authenticity of food claiming to be ‘halal’. In such situations, lay Muslims would take a more reactive approach and default to choosing a vegetarian, vegan or fish option in the absence of halal certified options being available: ‘*When you go out the choices are limited. You either go for the vegetarian dishes, or the fish option*’ (Participant 14, lay Muslim).

##### Sub-theme: Seeking purity within food

For faith leaders, the permissibility of food was judged not only by the way an animal was sacrificed but also by the pureness (tayyib) of the food. The terms ‘halal’ and ‘tayyib’ were used interchangeably and ‘organic’ and ‘wholesome’ foods were preferred: ‘*Islam teaches us to eat from the good and wholesome foods, “Qulubuna tayyibaat” [verse from the Qur’an] is about the good and wholesome foods. That is also a driver to engage in good food rather than eating junk. We think, if it's halal, it's fine. But there's so much more to it, is it wholesome? Is it pure? Everything halal isn’t necessarily good for you*’ (Participant 26, faith leader).

Muslim people expressed that intentionally nourishing one's body with ‘pure’ (tayyib) food was seen as an act of ‘worship’ (ibadah) and was a means of ‘feeding’ a spiritual connection with God: ‘*…eating has to be from a pure state, and if it's not, then that will affect your spirituality, to a certain degree that even when you call out in supplication it will not be answered because of that. [So] eat food that is organic, that is pure; eat from the best things possible. We believe as Muslims that food is one of those core things that not only affects you physically, but even affects your spirituality. It is very vital for us as believers to be careful of what we eat*’ (Participant 10, faith leader).

While faith leaders deemed the purity of food as a means of physical nourishment and worship, to lay Muslim participants their assessment of the purity of food was focused solely on the cleanliness of the meat and the draining of ‘impure’ blood when the animal is sacrificed: ‘*I am very strict on myself on making sure I am eating halal foods and staying away from foods that are prohibited for me to eat as a Muslim example, any animals that have not been slaughtered the Islamic way, saying* ‘*Bsimillah, Allahu Akbar*’*, before slaughtering and making sure the meat is clean. It is done in a respectful manner…the process in which they slaughter the meat is very clean… makes it so all the dirty blood leaves the meat.’* (Participant 1, lay Muslim)

#### Theme 2: Spheres of influence on food choice

Muslim peoples’ food choices were influenced by figures in Islam and their family setting. Two sub-themes describe the extent to which food choices are guided by influential figures in their lives, including ‘The Prophet Muhammed (PBUH) as a role model for food choice’ and ‘Islamic jurisprudence’.

##### Sub-theme: The Prophet Muhammed (PBUH) as a role model for food choice

The teachings of Islamic influential figures, specifically The Prophet Muhmmad (PBUH), influenced participants’ food choices. Food preference was guided by the Sunnah which are the traditions the Prophet Muhammad (PBUH) followed: ‘*I like dates and prefer to have olives in my diet. These are the things that I do sometimes because of the fact that the Holy Prophet used to do it. There is a level of affection as well involved in a sentimental way*.’ (Participant 7, faith leader)*.* There was a desire to adhere to what the Prophet Muhammad (PBUH) ate (e.g. milk, olives, figs, watermelon, grapes, pomegranate, vinegar, lamb shoulder, nigella seeds, gourd and pumpkin), to ‘emulate’ and feel closer to him: ‘*The Prophet SAW (sallallahu ‘alayhi wa sallam) used to like gourd. I’ve always enjoyed gourd and pumpkin. I was elated to think that I could be close to the Prophet, because I enjoyed this food. There's a whole selection of things I have not eaten, but that I want to eat because it's something in the tradition. And everything is about actually raising your consciousness around pleasing God*’ (Participant 27, faith leader).

Some foods recommended by the Sunnah of the Prophet Muhammad (PBUH) were eaten as they were considered to have health benefits that could treat illness; including honey because of its healing properties: ‘*Even when I'm ill my mom will make these concoctions of things like, turmeric. I know they're very cultural, but they also come from like the sunnah foods like honey… the benefits of them are also like ingrained so I think that's where sometimes culture and Islamic beliefs do come together’* (Participant 3, lay Muslim)

The desire to imitate the prophet's diet was also discussed in relation to meat. Participants expressed a preference to eating lamb shoulder, as well as making a conscious effort to follow his lifestyle (e.g. be ‘semi-vegetarian’), and consume more non-meat-based meals: ‘*And Islam has taught me how much meat I should eat. For example, the Prophet Muhammad SAW loved meat specifically the shoulder part, but he hardly ate it. So, you can see he was semi vegetarian. So, Islam also encourages us to eat vegetables more and less meat, eat less and pure*’ (Participant 23, faith leader).

##### Sub-theme: Islamic jurisprudence

Participants expressed a clear enactment of the ‘black and white’ rulings on foods and drinks that are halal and haram in Islam. However, there was an element of ‘grey’ in Islamic jurisprudence, which led to a difference of opinion amongst Sunni Muslims. This was especially illustrated through the variance in perspectives on the Islamic jurisprudence surrounding prawns: ‘*…there's the idea of makrūh but I still had it [prawns]*’ (Participant 22, lay Muslim). Muslim people exercised virtue and personal agency which influenced whether prawn was considered makrūh (disliked) or permissible (halal) and was, consequently, avoided or consumed based on their own independent reasoning: ‘*The school of thought I follow, it's just undesirable. I mean, it's unlawful. The actual prawns itself, not the flavouring’* (Participant 4, faith leader). Even within nuclear families, there were differences in interpretation of the Hanafi school of thought surrounding the consumption of prawns: ‘*My husband does not eat prawns. I eat it whenever my mom cooks it, but I don't bring it into the house*’ (Participant 23, faith leader). However, faith leaders felt ‘indebted’ to their teachers and followed their teacher's position on the consumption of prawns, regardless of their own interpretations: ‘*My teacher doesn't have the prawn, so I don't have it*’ (Participant 7, faith leader).

## Discussion

This research is the first published qualitative study to explore the influence of Islamic religiosity on food choices among both faith leaders and lay Muslims, allowing insight into how Islamic teaching influences nutrition. Both lay Muslim people and faith leaders shared a clear understanding of what foods are haram (impermissible) and halal (permissible). Choosing and consuming food that is halal was perceived as a requirement in the act of obedience to God, rather than a ‘food choice’ to make.

Halal certification, in the form of a visual logo or emblem, serves to inform and to reassure consumers that their products are halal compliant (Ambali and Bakar, 2014). In the present study, for Muslim people to determine whether food was ‘authentically’ halal, participants looked for a halal logo, deeming the HMC logo to be most trusted in the context of the UK. A qualitative study of Muslim people residing in Pakistan also found that a halal logo allowed participants to be confident of the food being halal and a reliable indicator of whether food is fit for consumption under Islamic law (Bukhari et al., 2019). In the absence of convincing verification of the status of a food being halal, Muslim people in this study would opt for a vegetarian option instead, which has not been reported in the literature before.

This study found that Muslim people trusted halal butchers and halal catering venues within their local community and distrusted supermarket chains. Similarly, consumer survey research has found that at the point of purchase, the majority of UK Muslims did not trust supermarket chains and only trust local shops run by Muslim owners and staff, which was reflected by most respondents (96%) purchasing their halal meat from local butchers (Ahmed, 2008). This study also reported that Muslim people preferred to travel to Muslim majority countries for vacations, to ensure their Islamic dietary requirements were catered for. Similarly, a qualitative study undertaken in the United Arab Emirates found that the availability of halal food affects travellers’ intentions to revisit a travel destination, and their length of stay (Mannaa, 2019).

Muslim people's food choices were ultimately role modelled by the food choices of the Prophet Muhammad (PBUH); an action referred to as ‘following the sunnah’. Hence, participants favoured eating ‘wholesome’ foods that were mentioned in Islamic scripture. Likewise, a qualitative study undertaken in Iran among university academics found that foods such as figs, olives, dates, honey, milk and olive oil were noted to be emphasised in the Qur’an and were particularly valued by participants, as they believed these to have positive influences on social and physical health (Salarvand and Pournia, 2014).

There was, however, some divergence in food choice between faith leaders and lay Muslims, suggesting that Muslim people who have undertaken further education of the religion of Islam have differential interpretations of the Qur’an compared to lay Muslim people. Notably, the permissibility of food was judged by faith leaders beyond food being halal, and instead favoured tayyib (pure) food (e.g. organic food), as this was seen to be an intentional act of ibadah (worship) that ‘fed’ a spiritual connection with God. This concept of tayyib in relation to food has not been reported in previous empirical research.

In addition, there was divergence between spheres of influence over faith leaders and lay Muslims in terms of what foods were considered makrūh (disliked) e.g. prawns. The Hanafi School of Islamic jurisprudence forbids consumption of seafood other than ‘fish’; however, there is disagreement among scholars about whether prawns/shrimp are considered ‘fish’ (Rind and Siddiqui, 2023). Family members were the main source of influence over the eating practices of lay Muslims. However, it was Islamic teachers who were influential over the eating practices of faith leaders. This difference in source of influence has not been reported in the literature before. It suggests that religious interpretation is passed down orally through the hierarchy of faith leaders and given precedence, which may explain further the Islamic jurisprudence seen around what foods are considered makrūh.

### Strengths and limitations

This study has several methodological strengths. Peer-review was performed to facilitate the development of the themes, enhancing the credibility of the findings, and data saturation was reached with a sample of 32 participants, ensuing transferability (Lincoln and Guba, 1986). The purposive sampling method ensured recruitment of equal numbers of faith leaders and lay Muslims, in a reasonably homogenous sample, allowing for a constant comparison analysis method to be employed with a solid basis for transferability (Boeije, 2002). However, there were also limitations. This study primarily consisted of Sunni Muslims with most identifying as South Asian. Therefore, the findings from the current study may not be transferrable to Muslims belonging to a different ethnicity or residing outside of the UK. Additionally, all participants were fluent English speakers hence the experiences of non-English Muslim speakers were not included in this study.

### Implications for health promotion

The findings from this study provide novel insights into the similarities and differences in food choice between Islamic faith leaders and lay Muslims, which can be leveraged by public health initiatives to co-develop and communicate evidence-based faith-informed behavioural dietary intervention to prevent ill health (World Health Organization, 2022). Islamic faith leaders are trusted and wield a great deal of influence within their community. Consequentially, they can promote willingness to take part in advocated lifestyle behaviours and allay concerns relating to faith appropriateness (Padela et al., 2011). In this way, faith leaders can act as ‘boundary spanners’ between healthcare and the local community (Long et al., 2013). Hence, involving faith leaders in faith-informed interventions could be a potential strategy to increase engagement in healthy eating behaviours in Muslim communities. Such an intervention, however, is an area requiring further research.

## Conclusion

This study has found that Islamic beliefs were influential in Muslim peoples’ decision-making around choice of food. The Prophet Muhammed (PBUH) was seen as a significant role model and Muslim people opted to eat foods outlined in Islamic scripture to emulate him. Muslim people were clear on foods that were halal or haram and would make food choices in line with this. However, lay Muslim people lacked confidence in determining whether meat provided by food outlets was authentically halal, in comparison to faith leaders who felt they could determine the authenticity of food claimed to be halal. There was, however, divergences in interpretations of Islamic scripture. Faith leaders viewed food to be tayyib (pure) if it provided health benefit, while lay Muslim people perceived purity as cleanliness. There was also jurisprudence amongst both lay Muslim people and faith leaders around whether the consumption of prawns was makrūh or halal, demonstrating a degree of personal virtue.

These findings offer an opportunity for healthcare professionals to promote and facilitate dietary education that is informed by Islam, to influence positive faith-informed health behaviours. Further qualitative research is required to examine the role of faith-informed dietary intervention in the Muslim community to prevent and treat long-term health conditions, and to explore a potential role for faith leaders in endorsing health promoting nutritional advice.

## Supplemental Material

sj-docx-1-nah-10.1177_02601060241244883 - Supplemental material for The influence of religiosity on food choice among British Muslims: A qualitative study Supplemental material, sj-docx-1-nah-10.1177_02601060241244883 for The influence of religiosity on food choice among British Muslims: A qualitative study by Umama Owais, Riya Patel and Sally Abbott in Nutrition and Health

sj-docx-2-nah-10.1177_02601060241244883 - Supplemental material for The influence of religiosity on food choice among British Muslims: A qualitative study Supplemental material, sj-docx-2-nah-10.1177_02601060241244883 for The influence of religiosity on food choice among British Muslims: A qualitative study by Umama Owais, Riya Patel and Sally Abbott in Nutrition and Health
