# Supplementary material for: The influence of religiosity on food choice among British Muslims: A qualitative study
Source: Nutr Health. 2024 Apr 3;31(2):749–56. doi: 10.1177/02601060241244883 (PMC12174622; doi:10.1177/02601060241244883)
Supplement: sj-docx-1-nah-10.1177_02601060241244883 - Supplemental material for The influence of religiosity on food choice among British Muslims: A qualitative study [file sj-docx-1-nah-10.1177_02601060241244883.docx]

**Supplement 1: Topic guide**

#### Introduction

Thank you for agreeing and taking the time to participate in this interview.

You are invited today so I can understand how Islamic beliefs influence the perspectives of Muslim people on their food selection and preferences.

There are no right or wrong answers, I am interested in your own opinions and experiences.

This interview will take around an hour. If, at any point, you would like to pause or stop the interview please, do not hesitate to let me know.

There are 10 questions in total. You are free to decline any question.

With your permission, I would like to record this interview, only on audio.

All responses from this interview will be kept strictly confidential. Only I will have access to the information.

If you have any questions regarding what I have just explained, please, feel free to ask.

The interview will begin now. May I turn on the recording?

#### Building Rapport

It would be great if you could start off by introducing yourself.

**Q**. Can you please tell me what made you participate in this study?

Did you have any personal interests, genuine curiosity or did you participate to help facilitate this research study?

#### Interview Questions

**Q1.** Can you tell me how your Islamic beliefs and values impact your eating habits?

**Probe:** (if impacted or not) could you please tell me why.

**Q2**. In what ways have your Islamic beliefs impacted your dietary preferences?

**Q3**. How has following a diet that aligns with your religious beliefs made it harder or easier for you?

**Prompt**: If easier- could you please tell me why it is easier?

If harder, why is that?

**Q4.** Is there a routine you follow when it comes to your diet?

**Prompt:** Are your reasons Islamic?

**Prompt:** If yes, have you noticed any positive changes?

Ramadan Routine:

**Any change you have noticed during Ramadan that you do not notice in the rest of the year?**

**Could you please describe the way you feel or the way your diet and how your relationship is with your diet during Ramadan compared to the rest of the year?**

If no, have you noticed any changes or experienced any difficulties due to not following an Islamic routine when it comes to your diet?

**Q5.** Has living in the UK as a Muslim effected/impacted your food selection?

**Prompt:** If yes- then, why, and how?

If no, why?

**Q6.** What do you avoid eating? What food do you sought out?

**Probe**: Why?

**Probe:** To what extent do you feel your reasons are linked to Islam? Could you please explain more?

**Q7.** Where do you usually purchase your grocery- a general convenience store or a halal supermarket?

**Prompt:** Could you please tell me why?

**Q8.** Is there anything specific you make sure to buy when grocery shopping?

**Q9.** How do you think your diet effects your health?

**Q10.** Do you believe your diet is healthy?

**Prompt:** “To what extent do you feel your diet is influenced by the teachings of Islam?”

#### Conclusion

Is there anything you would like to add or comment on regarding the topic?

Thank you for your time and sharing your thoughts today. It is very valuable and greatly appreciated.
